# Atrial structure and function in middle‐aged, physically‐active males and females: A cardiac magnetic resonance study

**DOI:** 10.1002/clc.23707

**Published:** 2021-09-01

**Authors:** Meghan Glibbery, Laura Banks, Mustafa A. Altaha, Robert F. Bentley, Kaja Konieczny, Andrew T. Yan, Paul Dorian, Djeven P. Deva, Jack M. Goodman, Kim A. Connelly

**Affiliations:** ^1^ Faculty of Kinesiology and Physical Education University of Toronto Toronto Canada; ^2^ Faculty of Medicine University of Toronto Toronto Canada; ^3^ Division of Cardiology St. Michael's Hospital Toronto Canada; ^4^ Keenan Research Centre, Li Ka Shing Knowledge Institute St. Michael's Hospital Toronto Canada; ^5^ Department of Medical Imaging, St. Michael's Hospital University of Toronto Toronto Canada; ^6^ Mount Sinai Hospital, Division of Cardiology University of Toronto Toronto Canada

**Keywords:** cardiac imaging, endurance exercise, middle‐age

## Abstract

Recent studies have reported on an association between endurance sport, atrial enlargement and the development of lone atrial fibrillation in younger, male cohorts. The atrial morphology and function of middle‐aged, physically‐active males and females have not been well studied. We hypothesized that middle‐aged males would demonstrate larger left atrium (LA) and right atrium (RA) volumes compared to females, but atrial function would not differ. LA and RA volume and function were evaluated at rest in healthy adults, using a standardized 3.0Tesla cardiac magnetic resonance protocol. Physical activity, medical history, and maximal oxygen consumption ($\dot{V}O_{2\text{peak}}$) were also assessed. Physically‐active, middle‐aged men (*n* = 60; 54 ± 5 years old) and women (*n* = 30; 54 ± 5 years old) completed this study. Males had a higher body mass index, systolic blood pressure, and $\dot{V}O_{2\text{peak}}$ than females (*p* < .05 for all), despite similar reported physical activity levels. Absolute and BSA and height‐indexed LA and RA maximum volumes were higher in males relative to females, despite no differences in ejection fractions (*p* < .05 for all). In multivariable regression, male sex *p* < .001) and $\dot{V}O_{2\text{peak}}$ (*p* = .004) were predictors of LA volume (model *R* ^2^ = 0.252), whereas $\dot{V}O_{2\text{peak}}$ (*p* < .001), male sex (*p* = .03), and RV EF (*p* < .05) were predictors of RA volume (model *R* ^2^ = 0.377). While middle‐aged males exhibited larger atrial volumes relative to females, larger, prospective studies are needed to explore the magnitude of physiologic atrial remodeling and functional adaptations in relation to phenotypic factors.

## 1 INTRODUCTION

Long‐term vigorous physical activity and exercise have been associated with cardiac morphological adaptations known as the “Athlete's Heart”.^1^ The extent of this remodeling in the atria has been well characterized in younger male and female elite endurance athletes using echocardiography.^2^, ^3^ However, there is paucity of literature describing atrial structure and function in middle‐aged physically‐active adults, despite the high participation rates of recreational to sub‐elite middle‐aged endurance athletes.^4^ This middle‐aged cohort exhibits cardiac morphological adaption in response to years of an intensive exercise‐training burden. We have recently characterized cardiac structure and function in this cohort using both electrocardiography^5^ and echocardiography,^6^ demonstrating significant and heterogeneous remodeling in the absence of increased atrial ectopy. A recent echocardiography study has reported an association between larger left atrial size and higher cardiorespiratory fitness in physically‐active adults across the lifespan.^7^ While echocardiography is a common first tool to evaluate cardiac structure, it underestimates cardiac volumes in comparison to the gold standard of cardiac magnetic resonance (CMR) imaging.^8^ While CMR has been applied to quantify atrial morphology in younger elite athletes,^9^, ^10^ only a single, small study has been completed in middle‐aged males consisting of 10 former elite athletes and five controls.^11^ The extent of atrial remodeling in this cohort may be particularly salient, as studies suggest a potential predilection for exercise induced cardiac remodeling and lone atrial fibrillation,^12^ particularly in male athletes.^13^ In fact, female athletes are less likely to develop atrial fibrillation at a younger age than men, and unlike men, there is no association between long‐standing exercise dose and risk of atrial fibrillation.^13^

Therefore, the primary objective of the current study was to characterize left atrium (LA) and right atrium (RA) morphology, as well as LA strain in middle‐aged, physically‐active adults. We hypothesized that males would demonstrate larger LA and RA volumes compared to females, but atrial function would not differ.

## 2 METHODS

### 2.1 General study procedures

This study was part of a larger investigation examining the cardiovascular phenotypic characteristics of healthy, physically‐active males and females.^5^, ^6^ All participants were between 45 and 65 years of age with a long‐standing history (>10 years) of physical activity participation. Exclusion criteria included a history of smoking, hypertension, diabetes mellitus, the use of cardio active drugs, or a history of chronic disease. Detailed medical and sport histories were obtained to confirm eligibility, and a physical examination was performed by a cardiologist. Participants were asked to abstain from caffeine (12 hours), alcohol (12 hours), and exercise (24 hours) prior to each study visit. The institutional research ethics board in accordance with the Declaration of Helsinki approved this study protocol. All participants provided written informed consent.

### 2.2 Exercise training history

All participants completed a two‐week exercise diary detailing the mode, duration, and intensity of each work out. A Likert scale was completed to determine the consistency of exercise training during the preceding 10 years and the training burden was determined by calculating both the h/week and the modality‐specific metabolic equivalent of a task (MET)·h/week of vigorous exercise.^14^

### 2.3 Resting blood pressure and maximal exercise testing

Resting seated blood pressure (BP) (BpTRU model BPM‐100, BpTRU Medical Devices, Coquitlam, BC, Canada) was measured in accordance with Canadian Hypertension guidelines^15^ prior to assessing cardiorespiratory fitness. A graded maximal exercise test was performed to exhaustion on a treadmill or cycle ergometer depending on the participant's primary exercise modality. Maximal oxygen consumption ($\dot{V}O_{2\text{peak}}$) was determined by breath‐by‐breath gas exchange, averaged every 20 seconds (Moxus Modular O_2_ system, Applied Electrochemistry Inc., Pittsburgh, PA).

### 2.4 CMR assessment and analysis

On a separate day, resting CMR in the supine position was performed to evaluate cardiac morphology. Images were acquired using a 3.0 Tesla magnet (Siemens MAGNETOM Skyra 3.0T with TIM and DOT technology) using a phased‐array cardiac coil and retrospective electrocardiographic gating, completed by one of two operators blinded to group and clinical status. Steady‐state free precession images were obtained during breath hold at end‐expiration. CINE images were acquired to obtain a contiguous short axis stack (slice thickness of 8 mm, no gaps), as well as long‐axis 4‐chamber and 2‐chamber images, as previously reported by our group.^16^ A total of 25 images per cardiac cycle were obtained at end‐tidal breath hold.

**FIGURE 1:**
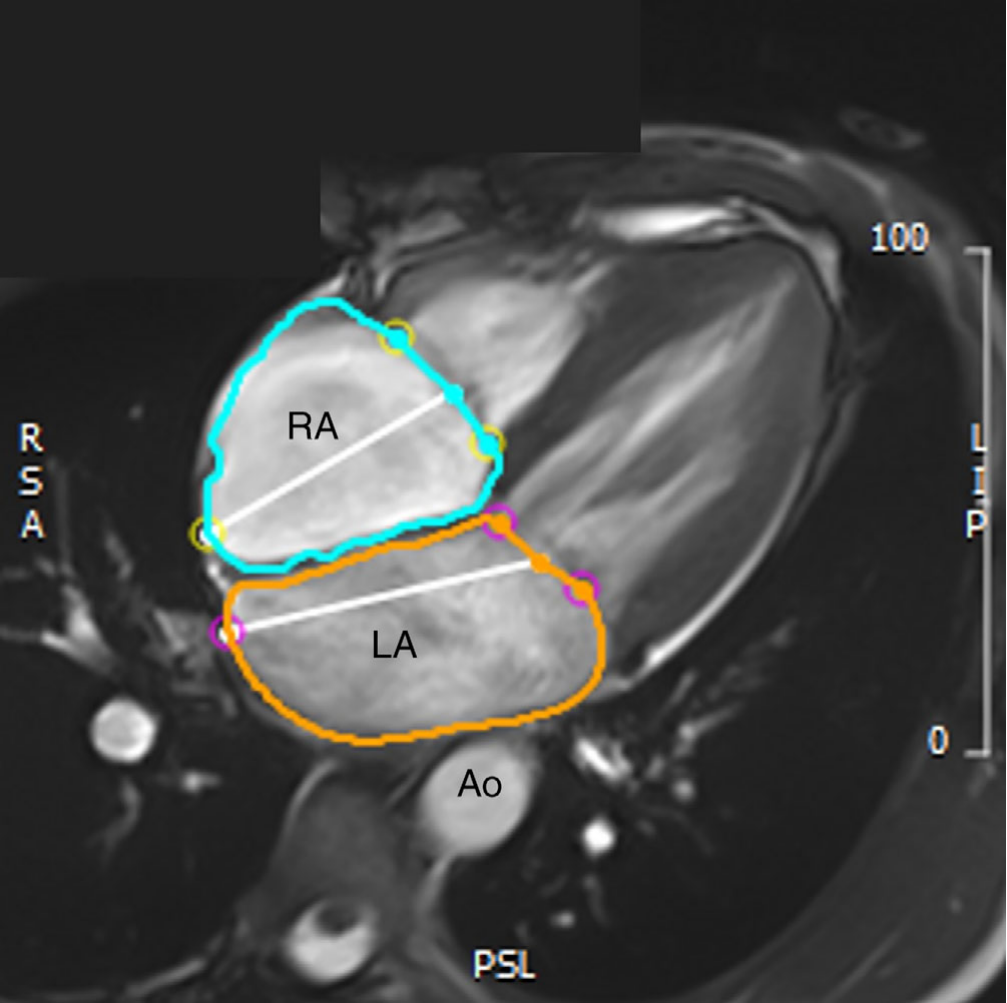
Example of the manual delineation of the left (orange) and right (light blue) atrium with cardiac magnetic resonance imaging (cMRI). The LA includes the left atrial appendage, but excludes the pulmonary vein, while the RA includes the right atrial appendage, while excludes the superior and inferior vena cava. Ao, aorta; LA, left atrium; RA, right atrium

CMR image analysis was performed with commercially available software (CVi42, Circle Cardiovascular Imaging, Calgary, AB, Canada) by a single blinded observer (MG). Inter‐observer repeatability was also performed with an additional blinded observer (MA). Intra‐ (*n* = 12) and inter‐observer (*n* = 5) repeatability demonstrated excellent intraclass correlation coefficients for the LA (0.99 intra‐rater, 0.97 inter‐rater) and RA (0.99 intra‐rater, 1.00 inter‐rater) volumes. Simpson's slice summation method was used for volumetric assessment (summation of outlined areas × slice thickness). Maximum and minimum volumes were manually traced with delineation of the atrial endocardial borders in all cardiac phases. The maximal LA and RA volumes were achieved immediately prior to ventricular diastole. The maximum volume was defined as the last image before opening of the mitral or tricuspid valve in the LA and RA, respectively. Accordingly, the minimal LA and RA volumes were defined as first image after closure of the mitral and tricuspid valves, immediately prior to ventricular systole. A straight line was drawn between the leading edge of the mitral or tricuspid valve annulus to determine the atrioventricular plane in the LA and RA, respectively (Figure 1). Atrial appendages were included, and the pulmonary veins and the superior and inferior vena cava were excluded. The relative degree of remodeling in the atrial and ventricular chambers was calculated using the following ratios: LA to RA volume indexed to height (LAmaxih/RAmaxih), LA to LV end‐diastolic volume indexed to height (LAmaxih/LVEDVih), and RA to RV end‐diastolic volume indexed to height (RAmaxih/RVEDVih). Maximal and minimal volumes were used to calculate atrial stroke volume (maximal volume – minimal volume = stroke volume [ml]) and ejection fraction (stroke volume/maximal volume × 100 = ejection fraction [%]). All cardiac volumes were indexed to height and body surface area (BSA), with height considered the most appropriate index for cardiac volumes in an athletic population.^17^ Additional normalized measures were calculated, including height^1.7^ and height^2.7^ .^18^ Atrial strain analysis was performed in a subset of male and female athletes with adequate image quality for the manual tracing of the LA endocardial borders in 2‐ and 4‐chamber long‐axis views (using LV end diastole as the reference phase). CVi42 software (Circle Cardiovascular Imaging, Inc, Calgary, Canada) was used to perform LA analyses. An automated tracking algorithm was applied, and manual adjustments were performed as needed to attain optimal wall tracking. We computed longitudinal atrial strain as (*L*_1_ − *L*_0_)/*L*_0_, where *L*_1_ is the change of atrial myocardial length throughout the atrial cycles and *L*_0_ is the resting (or reference) length in a relaxed state at diastasis (end of atrial diastole).

### 2.5 Statistical analysis

Normality was assessed visually with Q‐Q plots and quantitatively with a Shapiro–Wilk test. Independent sample t‐tests or Mann–Whitney U tests were conducted to evaluate differences between males and females. Data were presented as mean ± SD, unless otherwise specified. Stepwise multivariable regression was completed with $\dot{V}O_{2\text{peak}}$, sex and resting HR as predictors of LA volume, while $\dot{V}O_{2\text{peak}}$, sex, resting HR and RV EF were used as predictors of RA volume. Predictors were identified with univariate regression (included if *p* < .05), while sex was included a priori given we wanted to examine differences between sex. All assumptions of multivariable regression including normality of residuals, homoscedasticity and multi‐collinearity were met. All statistical analyses were completed using SPSS Statistics Software Version 20 (IBM Corp., Armonk, NY). Statistical significance was set at *p* < .05.

## 3 RESULTS

### 3.1 Participant demographics

Participant demographics (*n* = 90) are presented in Table 1. Two participants were not included in the analyses due to inadequate image quality. In brief, males had greater height (*p* < .001), weight (*p* < .001), body mass index (*p* = .005), and BSA (*p* < .001) compared to females. Males and females did not differ with respect to exercise training burden (both *p* = .113), but males did have a higher relative $\dot{V}O_{2\text{peak}}$ (*p* = .024). Males had a higher resting SBP (*p* = .002) and DBP (*p* = .003) compared to females.

**TABLE 1:** Participant demographics

	Male	Female
*N*	60	30
Age (years)	54 ± 5	54 ± 5
Height (m)	1.78 ± 0.05	1.64 ± 0.09*
Weight (kg)	77 ± 10	61 ± 11*
BMI (kg/m^2^)	24.3 ± 2.9	22.6 ± 3.4*
Vigorous exercise (hours/week)	7.0 ± 5.0	5.2 ± 3.6
Vigorous MET hours/week	73 ± 52	50 ± 36
Body surface area (m^2^)	1.95 ± 0.13	1.65 ± 0.17*
Resting SBP (mmHg)	117 ± 14	106 ± 13*
Resting DBP (mmHg)	75 ± 9	69 ± 9*
$\dot{V}O_{2\text{peak}}$ (ml/kg/min)	48.8 ± 8.6	43.8 ± 10.0*

*Note*: Data presented as mean ± standard deviation. * Denotes *p* < .05 for difference between male and female (*p* < .05).

Abbreviations: BMI, body mass index; DBP, diastolic blood pressure; SBP, systolic blood pressure; $\dot{V}O_{2\text{peak}}$, peak rate of oxygen consumption during exercise.

### 3.2 CMR atrial morphology and sex

Absolute atrial volumes as well as volumes indexed to both BSA and height are reported (Tables 2, 3). Males had larger absolute LA and RA volumes than females. LA volumes indexed to both height and BSA were also larger in males compared to females, as were indices of SV (Figure S1; all *p* < .05). LA EF was not different between males and females (*p* = .902). Similar observations were present for the RA, except that males had a greater RA EF (*p* = .001) compared to females and RA SV/BSA was not different (*p* = .210).

**TABLE 2:** Atrial volumes in physically‐active, middle‐aged adults

	Male	Female
*N*	60	30
LA max volume (ml)	124 ± 23	93 ± 22*
LA max/BSA (ml/m^2^)	63 ± 12	56 ± 13*
LA max/height (ml/m)	69 ± 13	57 ± 13*
LA min volume (ml)	64 ± 14	48 ± 12*
LA min/BSA (ml/m^2^)	33 ± 7	29 ± 7*
LA min/height (ml/m)	36 ± 8	29 ± 7*
LA SV (ml)	60 ± 13	45 ± 11*
LA SV/BSA (ml/m^2^)	31 ± 7	27 ± 7*
LA SV/height (ml/m)	34 ± 7	27 ± 7*
LA EF (%)	48 ± 6	48 ± 5
RA max volume (ml)	134 ± 31	96 ± 26*
RA max/BSA (ml/m^2^)	69 ± 17	58 ± 14*
RA max/height (ml/m)	75 ± 17	59 ± 14*
RA min volume (ml)	72 ± 20	47 ± 17*
RA min/BSA (ml/m^2^)	37 ± 11	29 ± 9*
RA min/height (ml/m)	41 ± 12	29 ± 9*
RA SV (ml)	61 ± 14	49 ± 12*
RA SV/BSA (ml/m^2^)	32 ± 8	29 ± 7
RA SV/height (ml/m)	34 ± 8	30 ± 7*
RA EF (%)	46 ± 6	51 ± 6*
LAmaxih/RAmaxih	0.96 ± 0.20	1.00 ± 0.21
LAmaxih/LVEDVih	0.61 ± 0.08	0.63 ± 0.12
RAmaxih/RVEDVih	0.61 ± 0.12	0.61 ± 0.10

*Note*: Data presented as mean ± standard deviation. * Denotes *p* < .05 for difference between male and female.

Abbreviations: BSA, body surface area; EF, ejection fraction; LA, left atrium; LAmaxih, left atrium maximum volume indexed to height; LV EDVih, left ventricle end diastolic volume indexed to height; Max, maximum; Min, minimum; RA, right atrium; RAmaxih, right atrium maximum volume indexed to height; RV EDVih, right ventricle maximum volume indexed to height; SV, stroke volume.

**TABLE 3:** Left atrial strain in physically‐active, middle‐aged adults

	Male	Female
N	34	16
4‐Chamber		
Peak radial strain (%)	40.9 ± 21.2	43.9 ± 23.5
Peak longitudinal strain (%)	−18.2 ± 4.9	−16.5 ± 12.9
Peak systolic radial strain rate (s^−1^)	2.4 ± 1.9	2.3 ± 1.2
Peak systolic longitudinal strain rate (s^−1^)	−1.1 ± 0.6	−1.1 ± 0.6
Peak diastolic radial strain rate (s^−1^)	−2.2 ± 1.5	−2.3 ± 2.4
Peak diastolic longitudinal strain rate (s^−1^)	1.0 ± 0.4	1.0 ± 0.7
2‐Chamber		
Peak radial strain (%)	44.3 ± 20.5	56.9 ± 14.8*
Peak longitudinal strain (%)	−17.9 ± 10.2	−21.3 ± 2.9
Peak systolic radial strain rate (s^−1^)	2.3 ± 2.1	3.4 ± 1.4*
Peak systolic longitudinal strain rate (s^−1^)	−1.1 ± 0.7	−1.5 ± 0.5*
Peak diastolic radial strain rate (s^−1^)	−2.4 ± 2.4	−2.1 ± 3.3
Peak diastolic longitudinal strain rate (s^−1^)	0.8 ± 0.8	1.0 ± 0.4

*Note*: Data presented as mean ± standard deviation. * Denotes difference from male versus female (*p* < .05).

### 3.3 CMR Assessment of right versus left atrial remodeling

The relative magnitude of atrial remodeling in the LA versus RA (LAmaxih/RAmaxih, *p* = 0.340), as well as atrial versus ventricular comparisons (LAmaxih/LVEDVih, *p* = .195; RAmaxih/RVEDVih, *p* = .966), did not differ between males and females (Table 2).

### 3.4 CMR assessment of LA strain

Among a subset of participants (males *n* = 34, females *n* = 16), 4‐chamber LA strain analysis revealed no sex differences. Strain imaging of the 2‐chamber view demonstrated worse peak radial strain (*p* = .005), peak systolic radial strain rate (*p* = .011) and peak systolic longitudinal strain rate (*p* = .013) in males compared to females (Table 3).

### 3.5 Predictors of left and right atrial remodeling

In univariate analysis, $\dot{V}O_{2\text{peak}}$ (*r* = 0.37, *p* < .001), resting HR (*r* = −0.31, *p* = 0.003) were independent predictors of LA max volume indexed to height. Point bi‐serial correlation demonstrated a positive association between male sex and LA max volume (*r* = 0.42, *p* < .001, Figure S1). Notably, SBP was not significantly associated with LA max volume (*r* = 0.11, *p* = .281). In multivariable regression analyses, $\dot{V}O_{2\text{peak}}$ (*β* = 0.231; *p* = .023), male sex (*β* = 0.333; *p* = .001), were significant predictors of LA max volume indexed to height; whereas, resting HR no longer remained a significant predictor (*β* = −0.19; *p* = .052) (Table 4; model *R* ^2^ = .286).

**TABLE 4:** Multivariable regression of factors associated with LA and RA max volumes

	Unstandardized coefficients		Standardized coefficients		
	*B*	Standard error	*β*	*T*	*p* value
Model LA max volume					
Sex	9.869	2.871	0.333	3.438	0.001
$\dot{V}O_{2\text{peak}}$	0.350	0.151	0.231	2.315	0.023
Heart rate	−0.294	0.149	−0.193	−1.968	0.052

	*R*	*R* ^2^	Adjusted *R* ^2^	Standard error of the estimate	*p* value
	0.535	.286	0.260	12.157	<0.001

	Unstandardized coefficients		Standardized coefficients		
	*B*	Standard error	*β*	*T*	*p* value
Model RA max volume					
Sex	9.956	3.469	0.261	2.870	0.005
$\dot{V}O_{2\text{peak}}$	0.645	0.177	0.332	3.652	<0.001
Heart rate	−0.434	0.174	−0.222	−2.494	0.015
RV EF	−0.708	0.371	−0.171	−1.907	0.060

	*R*	*R* ^2^	Adjusted *R* ^2^	Standard error of the estimate	*p* value
	0.649	0.421	0.393	14.146	<0.001

In univariate analysis $\dot{V}O_{2\text{peak}}$ (*r* = .49, *p* < .001), resting HR (*r* = −0.38, *p* < .001) RV EF (*r* = −0.35, *p* = .001, Figure S1) were independent predictors of RA max volume indexed to height. Notably, SBP was not significantly associated with RA max volume (*r* = −0.30, *p* = .780). Point bi‐serial correlation demonstrated a positive association between male sex and RA max volume (*r* = 0.43, *p* < .001). In multivariable regression analyses, $\dot{V}O_{2\text{peak}}$ (*β* = 0.332; *p* < .001), male sex (*β* = 0.261; *p* = .005), resting HR (*β* = −0.222; *p* = .015), were significant predictors of RA max volume indexed to height; whereas RV EF no longer remained a significant predictor (*β* = −0.171; *p* = .060) (Table 4; model *R* ^2^ = 0.421).

## 4 DISCUSSION

In the current study, we characterized atrial structure and function in healthy, middle‐aged adults. Our study cohort was unique in that it enabled the characterization of atrial remodeling across a spectrum of physical activity, ranging from middle‐aged males and females who performed recreational exercise (150 min/week of moderate‐vigorous physical activity) through to those who competed in recreational endurance sporting events. Males had larger atrial volumes than females with both LA and RA volumes being significantly and positively correlated to ventricular volumes, including left and right ventricular end diastolic volumes. These findings indicate proportional enlargement of all cardiac chambers in response to endurance training.

### 4.1 Atrial volumes and sex

Although the magnitude of cardiac remodeling varies considerably across individual athletes, male sex was the most significant predictor of LA maximum volume (indexed to height) in our multivariable regression analyses. Our findings support previous work where males have larger atrial volumes than females.^19^ In particular, Mosen et al.^9^ observed less atrial remodeling in female versus male endurance athletes when analyzing the ratios of LA and RA to total heart volume. In contrast, Letnes et al.^7^ also observed negligible differences in atrial volumes when adjusting for cardiorespiratory fitness.

The observation of larger atria in male athletes may be due to a number of underlying mechanisms. In the current study, males had higher systolic BP than females, which may contribute to atrial remodeling.^20^ In addition, a larger atria may be in part attributed to androgenic hormones that influence cardiac protein synthesis.^21^ Skeletal muscle mass, training volume, and plasma volume expansion may also influence the cardiovascular adaptations associated with training.^22^ Importantly, a recent randomized controlled trial demonstrated no sex differences in the magnitude of left atrial remodeling in previously sedentary adults who completed 24 months of high‐intensity training.^23^ Future investigation into the functional parameters of the LA and RA both at rest and during exercise may help to distinguish the influence of biological sex on atrial morphology and response to pathological conditions.

### 4.2 Atrial volumes and training

A recent meta‐analysis involving 16 echocardiographic studies of athletes (primarily males in high dynamic sports) reported that indexed LA volumes were unrelated to the type of sport performed.^24^ Our atrial morphology data in middle‐aged, physically‐active adults (ranging from recreationally‐active to sub‐elite) advances our understanding of atrial volumes among males and females who have engaged in long‐standing physical activity participation, as previous work often compared younger, male elite athletes to sedentary adults.^9^, ^11^, ^25^, ^26^, ^27^ Despite being the largest CMR study assessing atrial volumes in active middle aged persons to date, our sample size was not sufficient to rigorously assess the association between atrial volumes and physical activity modality.

The large variability of LA and RA volumes across our entire cohort suggests an equally wide and heterogeneous response to endurance training and atrial adaptation. McClean et al.^28^ similarly found no association between RA or LA volumes and years of athletic training; however, Wilhelm et al. ^29^ observed a positive correlation between LA volumes and lifetime training hours. The interaction of several factors, including genetics and those which contribute to the overall exercise training volume, not determined in the current study, likely significantly impacts this adaptation.

### 4.3 Atrial strain, endurance training, and sex

Our current CMR study findings revealed atrial enlargement and strain values within normal physiological range in middle‐aged, physically‐active adults. While our middle‐aged males had greater atrial enlargement than females, females exhibited superior atrial strain which may be secondary to their attenuated atrial remodeling response. Our findings expand upon previous echocardiographic studies focused primarily on younger, elite male athletes. In particular, a meta‐analysis of nine two‐dimensional speckle tracking echocardiographic studies observed that global LA longitudinal strain data was marginally lower in elite athletes relative to untrained athletes (mean ages ranging from 22 to 43 years of age).^30^ Notably, these studies primarily examined male athletes (7 of 9 studies had 100% male participants and 2 of 9 studies has 100% female participants), and therefore, were unable to evaluate the influence of endurance training and sex on atrial function. Recent work has also shown that worse atrial strain with higher atrial volumes in middle‐aged male athletes may be associated with atrial fibrillation.^31^ Whether this occurs as a result of structural, functional or electrical remodeling could not be determined with the current study.

### 4.4 Allometric scaling of the atria in athletes

The scaling of atrial volume warrants consideration. We reported various allometric scaling data in the present paper (Table S1). Some investigators advocate allometric scaling of cardiac volumes by height in the general population.^32^, ^33^ We chose to index cardiac volumes to height in the multivariable regression model as height may be a more appropriate index for cardiac volumes in a physically‐active population,^17^ although most commonly, atrial (and ventricular) volumes are scaled to BSA. It has also been argued that cardiac volumes should be scaled to fat‐free mass in athletic populations. The impact of scalar variables and its impact on athlete‐control comparisons of cardiac dimensions^18^ remains unresolved. While sex differences in lean body mass (LBM) may be amplified given the low‐fat mass present in both male and female endurance athletes, LBM is rarely analyzed in clinical research and was not assessed with the current study. These data suggest that further work is required to determine optimal scaling method for the atria, particularly in physically‐active cohorts.

### 4.5 Clinical implications

Whilst we demonstrated significantly more absolute and relative atrial enlargement in middle‐aged males relative to females, the clinical significance, such as a propensity to atrial arrhythmias in the future, remains unclear. A body of literature has emerged linking cardiac remodeling to lone AF, particularly in male relative to female athletes.^13^ Female athletes are less likely to develop atrial fibrillation at a younger age than men, and unlike men, there is no association between long‐standing exercise dose and risk of atrial fibrillation.^13^ This finding supports the emerging concept that absolute atrial size, as opposed to “indexed” atrial size, may be the prime determinant of the propensity toward AF.

Furthermore, recent CMR studies utilizing advanced atrial tissue characterization techniques to quantify scar tissue demonstrate a correlation between atrial fibrosis and AF risk.^34^ We were unable to perform high resolution atrial isotropic scans to assess the presence of atrial fibrosis. More studies are required to better characterize the atrial remodeling response at the tissue level in physically‐active cohorts, and to establish the association between “pathologic” remodeling of the atria, which includes absolute atrial size, markers of “excessive” or pathologic atrial fibrosis and the risk for lone atrial fibrillation in athletic cohorts.^12^

### 4.6 Limitations

Our study findings should be considered in light of the following limitations. We utilized a cross‐sectional design, which precludes any attribution of causality between atrial volumes and long‐term endurance exercise training. The self‐reporting of physical activity history may have introduced recall and response bias. We had a smaller cohort of physically‐active female participants; yet, we still observed that males have larger atrial volumes relative to females who performed similar levels of physical activity. The underrepresentation of females in clinical cardiovascular research remains widespread^35^ and future longitudinal research is required to understand sex differences in atrial structure and function in response to long‐term physical activity participation. Heterogeneity in atrial volumes may be even more pronounced if elite athletes and a sedentary cohort had been included.

## 5 CONCLUSIONS

The current study used gold standard CMR methods to measure atrial volumes and strain obtained from middle‐aged, physically‐active male and females. Although males had larger LA and RA volumes compared to the females, females exhibited superior atrial strain and strain rate values. Nonetheless, we observed significant heterogeneity in atrial structure among physically‐active, middle‐aged adults. Larger, prospective studies are needed to explore the magnitude of physiologic cardiac remodeling and functional adaptations in relation to atrial fibrosis, exercise training volume, and other phenotypic factors.

## CONFLICT OF INTEREST

The author declares that there is no conflict of interest that could be perceived as prejudicing the impartiality of the research reported.

## AUTHOR CONTRIBUTIONS

Meghan Glibbery, Laura Banks, Kaja Konieczny, Kim A. Connelly, Djeven P. Deva, Paul Dorian, Jack M. Goodman contributed to study design and data collection. Meghan Glibbery, Mustafa A. Altaha, Andrew T. Yan, Kim A. Connelly, Djeven P. Deva contributed to CMR protocol development and analyses. RFB performed statistical analyses. Laura Banks, RFB, Kim A. Connelly, Jack M. Goodman drafted the manuscript.

## Supporting information


**Table S1.** Indexed comparison of BSA, height, height^1.7^, height^2.7^.
**Figure S1**. Correlation between left atrial (LA) and right atrial (RA) volumes and $\dot{V}O_{2\text{peak}}$ by sex (Panel A: females; Panel B: males). LAmaxih, left atrial maximum volume indexed to height; RAmaxih, right atrial maximal volume indexed to height; $\dot{V}O_{2\text{peak}}$, peak oxygen consumption.

## Data Availability

Author elects to not share data.

## References

[1] Maron BJ . Structural features of the athlete heart as defined by echocardiography. J Am Coll Cardiol. 1986;7:190‐203. 2934463 10.1016/s0735-1097(86)80282-0

[2] D'Ascenzi F , Pelliccia A , Solari M , et al. Normative reference values of right heart in competitive athletes: a systematic review and meta‐analysis. J Am Soc Echocardiogr. 2017;30:845, e842‐858. 28865556 10.1016/j.echo.2017.06.013

[3] Iskandar A , Mujtaba MT , Thompson PD . Left atrium size in elite athletes. JACC Cardiovasc Imaging. 2015;8:753‐762. 26093921 10.1016/j.jcmg.2014.12.032

[4] USA R . 2016 State of the Sport ‐ U.S. Road Race Trends 2016.

[5] Konieczny K , Banks L , Osman W , et al. Prolonged P wave duration is associated with right atrial dimensions, but not atrial arrhythmias, in middle‐aged endurance athletes. J Electrocardiol. 2019;56:115‐120. 31394411 10.1016/j.jelectrocard.2019.07.002

[6] Banks L , Bentley RF , Currie KD , et al. Cardiac remodeling in middle‐aged endurance athletes and recreationally active individuals: challenges in defining the “Athlete's heart”. J Am Soc Echocardiogr. 2019;33(2):247‐249. 31812548 10.1016/j.echo.2019.09.014

[7] Letnes JM , Nes B , Vaardal‐Lunde K , et al. Left atrial volume, cardiorespiratory fitness, and diastolic function in healthy individuals: the HUNT study, Norway. J Am Heart Assoc. 2020;9:e014682. 31986991 10.1161/JAHA.119.014682 PMC7033857

[8] Grothues F , Smith GC , Moon JC , et al. Comparison of interstudy reproducibility of cardiovascular magnetic resonance with two‐dimensional echocardiography in normal subjects and in patients with heart failure or left ventricular hypertrophy. Am J Cardiol. 2002;90:29‐34. 12088775 10.1016/s0002-9149(02)02381-0

[9] Mosen H , Steding‐Ehrenborg K . Atrial remodelling is less pronounced in female endurance‐trained athletes compared with that in male athletes. Scand Cardiovasc J. 2014;48:20‐26. 24279839 10.3109/14017431.2013.860234

[10] Scharf M , Brem MH , Wilhelm M , Schoepf UJ , Uder M , Lell MM . Atrial and ventricular functional and structural adaptations of the heart in elite triathletes assessed with cardiac MR imaging. Radiology. 2010;257:71‐79. 20807850 10.1148/radiol.10092377

[11] Sanchis‐Gomar F , Garatachea N , Catalan P , Lopez‐Ramon M , Lucia A , Serrano‐Ostariz E . LA size in former elite athletes. JACC Cardiovasc Imaging. 2016;9:630‐632. 10.1016/j.jcmg.2015.08.023 26897686

[12] Goodman JM , Banks L , Connelly KA , Yan AT , Backx PH , Dorian P . Excessive exercise in endurance athletes: Is atrial fibrillation a possible consequence? Appl Physiol Nutr Metab. 2018;43:973‐976. 29842800 10.1139/apnm-2017-0764

[13] Svedberg N , Sundstrom J , James S , Hallmarker U , Hambraeus K , Andersen K . Long‐term incidence of atrial fibrillation and stroke among cross‐country skiers. Circulation. 2019;140:910‐920. 31446766 10.1161/CIRCULATIONAHA.118.039461

[14] Pescatello LS . ACSM's Guidelines for Exercise Testing and Prescription. 9th ed. Philadelphia: Wolters Kluwer/Lippincott Williams & Wilkins Health; 2014.

[15] Harris KC , Benoit G , Dionne J , et al. Hypertension Canada's 2016 canadian hypertension education program guidelines for blood pressure measurement, diagnosis, and assessment of risk of pediatric hypertension. Can J Cardiol. 2016;32:589‐597. 27118292 10.1016/j.cjca.2016.02.075

[16] Verma S , Mazer CD , Yan AT , et al. Effect of empagliflozin on left ventricular mass in patients with type 2 diabetes and coronary artery disease: the EMPA‐HEART CardioLink‐6 randomized clinical trial. Circulation. 2019;140(21):1693‐1702. 31434508 10.1161/CIRCULATIONAHA.119.042375

[17] Dewey FE , Rosenthal D , Murphy DJ Jr , Froelicher VF , Ashley EA . Does size matter? Clinical applications of scaling cardiac size and function for body size. Circulation. 2008;117:2279‐2287. 18443249 10.1161/CIRCULATIONAHA.107.736785

[18] George K , Sharma S , Batterham A , Whyte G , McKenna W . Allometric analysis of the association between cardiac dimensions and body size variables in 464 junior athletes. Clin Sci (Lond). 2001;100:47‐54. 11115417

[19] Kawel‐Boehm N , Maceira A , Valsangiacomo‐Buechel ER , et al. Normal values for cardiovascular magnetic resonance in adults and children. J Cardiovasc Magn Reson. 2015;17:29. 25928314 10.1186/s12968-015-0111-7 PMC4403942

[20] Oliver W , Matthews G , Ayers CR , et al. Factors associated with left atrial remodeling in the general population. Circ Cardiovasc Imaging. 2017;10(2):e005047. 28153949 10.1161/CIRCIMAGING.116.005047 PMC5302131

[21] Fagard R , Saddi R . Ferritin structure and biosynthesis. Biochimie. 1977;59:765‐773. 341990 10.1016/s0300-9084(77)80205-8

[22] D'Ascenzi F , Pelliccia A , Natali BM , et al. Morphological and functional adaptation of left and right atria induced by training in highly trained female athletes. Circ Cardiovasc Imaging. 2014;7:222‐229. 24470314 10.1161/CIRCIMAGING.113.001345

[23] McNamara DA , Aiad N , Howden E , et al. Left atrial electromechanical remodeling following 2 years of high‐intensity exercise training in sedentary middle‐aged adults. Circulation. 2019;139:1507‐1516. 30586729 10.1161/CIRCULATIONAHA.118.037615 PMC6422706

[24] Cuspidi C , Sala C , Tadic M , et al. Left atrial volume in elite athletes: a meta‐analysis of echocardiographic studies. Scand J Med Sci Sports. 2019;29:922‐932. 30866082 10.1111/sms.13416

[25] Scharhag J , Thunenkotter T , Urhausen A , Schneider G , Kindermann W . Echocardiography of the right ventricle in athlete's heart and hearts of normal size compared to magnetic resonance imaging: which measurements should be applied in athletes? Int J Sports Med. 2010;31:58‐64. 20029739 10.1055/s-0029-1241209

[26] Gjerdalen GF , Hisdal J , Solberg EE , Andersen TE , Radunovic Z , Steine K . Atrial size and function in athletes. Int J Sports Med. 2015;36:1170‐1176. 26509381 10.1055/s-0035-1555780

[27] Sanchis L . Sanz‐de La Garza M, Bijnens B, et al. gender influence on the adaptation of atrial performance to training. Eur J Sport Sci. 2017;17:720‐726. 28287029 10.1080/17461391.2017.1294620

[28] McClean G , George K , Lord R , et al. Chronic adaptation of atrial structure and function in elite male athletes. Eur Heart J Cardiovasc Imaging. 2015;16:417‐422. 25368211 10.1093/ehjci/jeu215

[29] Wilhelm M , Roten L , Tanner H , Wilhelm I , Schmid JP , Saner H . Atrial remodeling, autonomic tone, and lifetime training hours in nonelite athletes. Am J Cardiol. 2011;108:580‐585. 21658663 10.1016/j.amjcard.2011.03.086

[30] Cuspidi C , Tadic M , Sala C , Gherbesi E , Grassi G , Mancia G . Left atrial function in elite athletes: a meta‐analysis of two‐dimensional speckle tracking echocardiographic studies. Clin Cardiol. 2019;42:579‐587. 30907013 10.1002/clc.23180 PMC6523010

[31] Trivedi SJ , Claessen G , Stefani L , et al. Differing mechanisms of atrial fibrillation in athletes and non‐athletes: alterations in atrial structure and function. Eur Heart J Cardiovasc Imaging. 2020;21:1374‐1383. 32757003 10.1093/ehjci/jeaa183

[32] George KP , Batterham AM , Jones B . The impact of scalar variable and process on athlete‐control comparisons of cardiac dimensions. Med Sci Sports Exerc. 1998;30:824‐830. 9624638 10.1097/00005768-199806000-00008

[33] Kuznetsova T , Haddad F , Tikhonoff V , et al. Impact and pitfalls of scaling of left ventricular and atrial structure in population‐based studies. J Hypertens. 2016;34:1186‐1194. 27035735 10.1097/HJH.0000000000000922

[34] Marrouche NF , Wilber D , Hindricks G , et al. Association of atrial tissue fibrosis identified by delayed enhancement MRI and atrial fibrillation catheter ablation: the DECAAF study. JAMA. 2014;311:498‐506. 24496537 10.1001/jama.2014.3

[35] Gong IY , Tan NS , Ali SH , et al. Temporal trends of women enrollment in major cardiovascular randomized clinical trials. Can J Cardiol. 2019;35:653‐660. 31030866 10.1016/j.cjca.2019.01.010

