# Supplementary material for: Atrial structure and function in middle‐aged, physically‐active males and females: A cardiac magnetic resonance study
Source: Clin Cardiol. 2021 Sep 1;44(10):1467–74. doi: 10.1002/clc.23707 (PMC8495091; doi:10.1002/clc.23707)
Supplement: Supplementary file 1 — Table S1. Indexed comparison of BSA, height, height1.7, height2.7. Figure S1. Correlation between left atrial (LA) and right atrial (RA) volumes and V˙O2peak by sex (Panel A: females; Panel B: males). LAmaxih, left atrial maximum volume indexed to height; RAmaxih, right atrial maximal volume indexed to height; V˙O2peak, peak oxygen consumption. [file CLC-44-1467-s001.docx]

Supplementary Table 1: Indexed comparison of BSA, height, height^1.7^, height^2.7^

|  | Male | Female |
| --- | --- | --- |
| *N* | 60 | 30 |
| LA max/BSA (ml/m^2^) | 63 ± 12 | 56 ± 13* |
| LA max/height (ml/m) | 69 ± 13 | 57 ± 13* |
| LA max/height^1.7^ (ml/m^1.7^) | 46 ± 9 | 40 ± 9* |
| LA max/height^2.7^ (ml/m^2.7^) | 26 ± 5 | 25 ± 6 |
|  |  |  |
| LA min/BSA (ml/m^2^) | 33 ± 7 | 29 ± 7* |
| LA min/height (ml/m) | 36 ± 8 | 29 ± 7* |
| LA min/height^1.7^ (ml/m^1.7^) | 24 ± 5 | 21 ± 5* |
| LA min/height^2.7^ (ml/m^2.7^) | 13 ± 3 | 13 ± 3 |
|  |  |  |
| LA SV/BSA (ml/m^2^) | 31 ± 7 | 27 ± 7* |
| LA SV/height (ml/m) | 34 ± 7 | 27 ± 7* |
| LA SV/ height^1.7^ (ml/m^1.7^) | 22 ± 5 | 19 ± 5* |
| LA SV/ height^2.7^ (ml/m^2.7^) | 13 ± 3 | 12 ± 3 |
|  |  |  |
| RA max/BSA (ml/m^2^) | 69 ± 17 | 58 ± 14* |
| RA max/height (ml/m) | 75 ± 17 | 59 ± 14* |
| RA max/height^1.7^ (ml/m^1.7^) | 50 ± 12 | 41 ± 10* |
| RA max/height^2.7^ (ml/m^2.7^) | 28 ± 7 | 25 ± 6 |
|  |  |  |
| RA min/BSA (ml/m^2^) | 37 ± 11 | 29 ± 9* |
| RA min/height (ml/m) | 41 ± 12 | 29 ± 9* |
| RA min/height^1.7^ (ml/m^1.7^) | 27 ± 8 | 20 ± 6* |
| RA min/height^2.7^ (ml/m^2.7^) | 15 ± 4 | 12 ± 4* |
|  |  |  |
| RA SV/BSA (ml/m^2^) | 32 ± 8 | 29 ± 7 |
| RA SV/height (ml/m) | 34 ± 8 | 30 ± 7* |
| RA SV/height^1.7^ (ml/m^1.7^) | 23 ± 5 | 21 ± 5 |
| RA SV/height^2.7^ (ml/m^2.7^) | 13 ± 3 | 13 ± 3 |
|  |  |  |

Data presented as mean ± standard deviation. LA, left atrium; Max, maximum; BSA, body surface area; Min, minimum; SV, stroke volume; RA, right atrium. * Denotes p<0.05 for difference between male and female.

Supplementary Figure 1. Correlation between left atrial (LA) and right atrial (RA) volumes and $\dot{V}$O_2peak_ by sex (panel A: females; panel B: males). LAmaxih, left atrial maximum volume indexed to height; RAmaxih, right atrial maximal volume indexed to height; $\dot{V}$O_2peak_, peak oxygen consumption.
